# Image-based phenotyping of plant disease symptoms

**DOI:** 10.3389/fpls.2014.00734

**Published:** 2015-01-05

**Authors:** Andrew M. Mutka, Rebecca S. Bart

**Affiliations:** Donald Danforth Plant Science Center, Saint Louis, MO, USA

**Keywords:** plant disease, phenotyping, imaging, pathogen, host

## Abstract

Plant diseases cause significant reductions in agricultural productivity worldwide. Disease symptoms have deleterious effects on the growth and development of crop plants, limiting yields and making agricultural products unfit for consumption. For many plant–pathogen systems, we lack knowledge of the physiological mechanisms that link pathogen infection and the production of disease symptoms in the host. A variety of quantitative high-throughput image-based methods for phenotyping plant growth and development are currently being developed. These methods range from detailed analysis of a single plant over time to broad assessment of the crop canopy for thousands of plants in a field and employ a wide variety of imaging technologies. Application of these methods to the study of plant disease offers the ability to study quantitatively how host physiology is altered by pathogen infection. These approaches have the potential to provide insight into the physiological mechanisms underlying disease symptom development. Furthermore, imaging techniques that detect the electromagnetic spectrum outside of visible light allow us to quantify disease symptoms that are not visible by eye, increasing the range of symptoms we can observe and potentially allowing for earlier and more thorough symptom detection. In this review, we summarize current progress in plant disease phenotyping and suggest future directions that will accelerate the development of resistant crop varieties.

## INTRODUCTION

Plant disease is a major threat for global agriculture, accounting for at least a 10% reduction in global yields (Strange and Scott, 2005). Subsistence farmers are the most at risk from plant diseases, as they often have limited resources to deal with outbreaks. Most resistant crop varieties have been developed through breeding with resistance (*R*) genes. These *R* genes typically recognize the activity of pathogen virulence factors to induce strong resistance responses (Dangl et al., 2013). This approach has been successful in some cases, but often resistance is quickly lost, which is thought to be due to rapid pathogen evolution (Kunkeaw et al., 2010; Dangl et al., 2013). An in depth understanding of the molecular interplay between hosts and their pathogens will guide the development of durable resistance strategies for crop protection. However, research aimed at identifying and characterizing the genetic determinants of host–pathogen interactions is often obfuscated by functional redundancy, especially on the side of the pathogen. In essence, single gene mutants often lack dramatic phenotypes and are therefore difficult to study.

The advancement of genome and transcriptome sequencing technologies has helped address the above challenge by making it possible to study the genetic diversity present in both plant hosts and pathogens (Bart et al., 2012; Berkman et al., 2012). Currently, much research is aimed at characterizing this diversity with the goal of exploiting it for the development of crops with durable resistance. The ability to translate knowledge of genomic variants into desired resistance phenotypes would be aided by a more complete understanding of the relationship between genotype and phenotype in plants. Nonetheless, our ability to study plant phenomics has not progressed at the same rate as our ability to sequence genomes and transcriptomes. In recent years, there has been considerable interest and progress in the development of platforms for quantitative, high-throughput plant phenotyping (Furbank and Tester, 2011; Dhondt et al., 2013; Fiorani and Schurr, 2013; Araus and Cairns, 2014; Granier and Vile, 2014).

The field of machine vision develops tools that perform automated image acquisition and analysis to understand and quantify aspects of a scene. Increasingly, these approaches are being applied to studies of plant growth and development (Spalding and Miller, 2013). Image-based phenotyping methods offer a range of advantages. They are non-destructive, meaning that phenotypic data can be collected from the same organism over the course of a long experiment. They are also amenable to automation, making it feasible to study large sample sizes for increased statistical power. Particularly important for plant–pathogen interactions, imaging can detect spatial patterns of heterogeneity and allows for visualization of localized responses, which may be difficult to determine with other methods. Additionally, various imaging techniques, such as hyperspectral and thermal imaging, collect data that cannot be visualized with the human eye.

In this review, we discuss the application of image-based phenotyping methods that have the potential to dramatically enhance our ability to characterize plant disease phenotypes. The techniques discussed have the potential to increase the dimensions at which an interaction is investigated and can be deployed in a high-throughput manner. If further developed and employed correctly, phenomics will increase our understanding of host–pathogen interactions and facilitate the development of durable resistance strategies. The methods for phenotyping disease symptoms can be broadly divided into (1) data collection and (2) data analysis, both of which deserve careful attention in experimental design. We hope that this review will bring plant phenomics to the attention of the host–microbe community and inspire further development of these promising technologies.

## IMAGE-BASED METHODS FOR ASSESSMENT OF PLANT DISEASE SYMPTOMS

### DATA COLLECTION

#### Visible light imaging

Traditionally, plant disease severity is scored with visual inspection of plant tissue by trained raters, who categorize disease severity according to a discrete scale (Bock et al., 2010). While this approach has been refined over many years and many crop systems, it still is plagued by inherent pitfalls that reduce the reliability of disease estimates. Substantial variation is observed both between individual raters and between different assessments by a single rater (Nutter et al., 1993; Bock et al., 2008, 2010). Accurate visual estimates are particularly difficult to achieve with certain types of disease symptoms, such as small, evenly spaced lesions (Bock et al., 2010). Additionally, because of the cost of labor and time needed to perform visual assessments of disease, the number of time points from which data can be sampled is limited.

The use of automated, high-throughput digital imaging in plant disease phenotyping allows for collection of data at numerous time points, produces images from which quantitative phenotypic data can be derived, and improves reproducibility of experiments. Many different phenotypic measurements can be obtained from image data. For studies of plant growth and development, these measurements may be plant height or biomass. For studies of plant disease or other stresses, percent leaf area covered with symptoms or changes in photosynthetic responses can be derived from images. The scale of imaging systems can vary greatly. For example, image-based phenotyping of a small number of plants can be performed with inexpensive, portable systems, such as the Raspberry Pi computer and camera^1^. On a larger scale, high-throughput phenotyping systems, such as the Bellwether Foundation Phenotyping Facility at the Donald Danforth Plant Science Center^2^ and the Australian Plant Phenomics Facility^3^, can perform automated imaging of hundreds or thousands of plants.

Studies with a variety of pathogens have found that image-based phenotyping produces more accurate and precise results than can be obtained with visual assessments of disease, and allows for exploration of more dimensions of disease phenotypes. For example, imaging of wheat leaves infected with strains of the fungal pathogen *Zymoseptoria tritici* was compared to traditional visual assessment methods (Stewart and McDonald, 2014). This fungus causes septoria wheat blotch, which is characterized by chlorosis, necrotic lesions, and fungal fruiting bodies called pycnidia. Typical visual disease assessments rely on estimates of percent of leaf area covered by pycnidia or lesions. Since pycnidia are small, accurate estimates of pycnidia cover are difficult to make, especially when they are numerous. Stewart and McDonald (2014) used automated image analysis of infected wheat leaves to analyze disease symptoms caused by *Z. tritici*. This approach allowed them to quantify pycnidia size and density, along with other traits, which would not have been possible with visual assessment alone. Thus, image-based phenotyping can greatly enhance the data available for characterizing plant disease.

Since variability between different ratings and between different raters is a limitation for visual disease assessment, image-based phenotyping offers the potential to improve reproducibility and sensitivity of disease quantification. Bock et al. (2008) examined citrus canker disease symptoms on grapefruit leaves caused by the bacterium *Xanthomonas axonopodis* pv. *citri* using digital imaging. The authors found that automated image analysis was more reproducible than visual assessments over multiple measurements. Another study found that image analysis enhanced the ability to distinguish between genotypes with different levels of disease severity. Xie et al. (2012) studied common bacterial blight caused by *Xanthomonas* spp. on two different genotypes of bean. While for this particular disease all measurements of the disease were reproducible between different assessments, image analysis was better able to distinguish between different disease susceptibility levels on different genotypes. The authors concluded that image analysis is more useful for investigating the quantitative genetics of disease resistance for this system. Thus, analysis of plant disease symptoms with visible light imaging has been shown to have a variety of benefits, which depend on the plant–pathogen system being analyzed.

**FIGURE 1:**
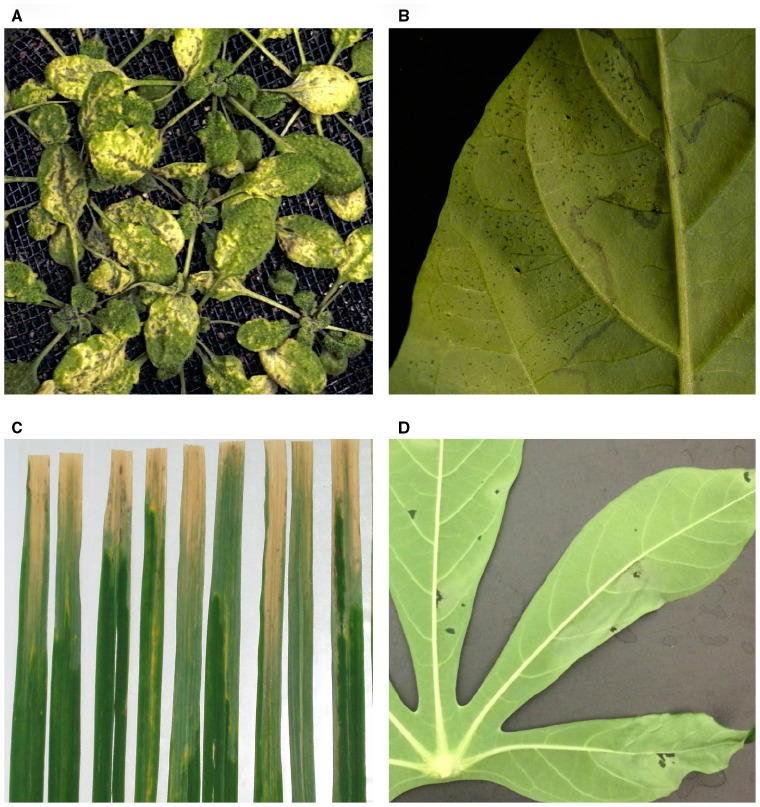
**Examples of disease symptoms caused by bacterial plant pathogens.** All photos were taken by the authors. **(A)** *Pseudomonas syringae* infection on *Arabidopsis thaliana* with gray water-soaked lesions surrounded by chlorosis. **(B)** Early-stage *Xanthomonas euvesicatoria* infection on pepper with small water-soaked lesions. **(C)** *Xanthomonas oryzae* pv. oryzae infection on rice with grayish green water-soaked lesions coalescing into yellow streaks. **(D)** *Xanthomonas axonopodis* pv. *manihotis* infection on cassava with dark water-soaked lesions that are spreading and leading to leaf wilt.

The terminology used to describe plant disease symptoms often does not reflect the symptom variety that is seen across different systems. For example, various symptoms caused by bacterial plant pathogens on different hosts are described as water-soaked lesions. However, these lesions vary greatly in size, shape, and color (Figure 1). Phenotyping studies must be clear in defining the symptoms that are being measured. To enable comparability between systems and storage of phenotypic data in databases, a standardized set of nomenclature for disease symptoms would be beneficial, similar to what was done for plant anatomy and morphology with the Plant Structure Ontology (Ilic et al., 2007). Some efforts to achieve this for plant–pathogen interactions are currently in development (Walls et al., 2012; Rodriguez Iglesias et al., 2013). Additionally, the use of experimental controls, such as internal color standards, would improve comparability between different imaging platforms.

#### Chlorophyll fluorescence imaging

Visible disease symptoms do not provide all of the available information about plant health and may not be the best indicator for plant disease severity, especially early during infection. Plants that are experiencing biotic and abiotic stresses exhibit changes in chlorophyll fluorescence emission (Baker, 2008). Under normal conditions, most chlorophyll fluorescence is emitted from photosystem II (PSII) at 685 nm (Rolfe and Scholes, 2010). When plants experience stress, this results in altered patterns of chlorophyll fluorescence emission, which can be observed with fluorescence imaging.

One of the most widely studied parameters based on chlorophyll fluorescence is *F*_v_/*F*_m_, also known as the maximum quantum efficiency of PSII (Baker, 2008). This parameter is calculated from *F*_m_, the maximum fluorescence of a dark-adapted leaf, and *F*_v_, the difference between *F*_m_ and the minimum fluorescence from dark-adapted leaf (*F*_0_). While non-stressed plants maintain a consistent *F*_v_/*F*_m_ value, various studies have shown that plants experiencing biotic or abiotic stresses have decreased *F*_v_/*F*_m_ values (Bonfig et al., 2006; Jansen et al., 2009; Bauriegel et al., 2011; Rousseau et al., 2013; Bauriegel and Herppich, 2014). Changes in this parameter occur before visible disease symptoms occur (Bonfig et al., 2006; Rolfe and Scholes, 2010). Studies of *Pseudomonas syringae* infection on *Arabidopsis thaliana* suggest this pathogen has direct impacts on PSII function (Bonfig et al., 2006). Thus, imaging of chlorophyll fluorescence can provide added value relative to standard visualization of symptoms.

In addition to *F*_v_/*F*_m_, other photosynthetic parameters have been studied in the context of plant disease. Non-photochemical quenching (NPQ or q_*N*_) is a measure of energy that is dissipated as heat from photosynthetic reaction centers. Rodriguez-Moreno et al. (2008) observed that NPQ goes up initially during *P. syringae* infection on bean plants, but then is decreased at the later stages of infection. Furthermore, NPQ was shown to have more leaf-to-leaf variation and different effects resulting from physiological changes and tissue death for *P. syringae* infection on *A. thaliana* (Berger et al., 2007). Thus, NPQ has a more complex relationship with plant stress than that of *F*_v_/*F*_m_. Other photosynthetic parameters that have been studied in context of disease are *Φ*_*PSII*_, the effective quantum yield of PSII [also known as *Y*(*II*) or *F*_q_^′^/*F*_m_^′^], and relative electron transport rate (ETR; Bonfig et al., 2006; Baker, 2008; Rolfe and Scholes, 2010). *Φ*_*PSII*_ is a parameter that is similar to *F*_v_/*F*_m_ but measured in illuminated conditions, and ETR is calculated from *Φ*_*PSII*_ and the amount of photosynthetically active light that is absorbed by the leaf (Rolfe and Scholes, 2010). These parameters exhibit a wide range of responses for different pathogen–host systems (Bonfig et al., 2006; Scholes and Rolfe, 2009; Rolfe and Scholes, 2010). A few studies have used a “combinatorial” approach, measuring several photosynthetic parameters at once, including those without explicit physiological meaning (Matous et al., 2006; Berger et al., 2007). While these novel parameters may exhibit correlations with pathogen infection, their physiological relevance is unknown (Rolfe and Scholes, 2010).

Overall, chlorophyll fluorescence imaging has benefits in providing physiological information about plant health, which in some cases allows for detection of disease prior to visible symptoms appearing. However, the technical challenges of this type of imaging, such as dark-adaptation for *F*_v_/*F*_m_ measurements, makes it difficult to translate to agricultural fields. Nonetheless, researchers are developing methods to move this technology outside the laboratory, such as shaded imaging stations that allow dark-adapted measurements to be taken in the field (Bauriegel et al., 2011; Bauriegel and Herppich, 2014). Regardless of whether this technique will prove effective for disease monitoring in the field, fluorescence imaging will continue to be an important tool for studying plant disease in the laboratory.

#### Hyperspectral imaging

Hyperspectral imaging is a relatively new technology that involves the acquisition of electromagnetic spectra at every pixel in an image, thus combining spatial and spectral information (Bock et al., 2010). Since hyperspectral images have two spatial dimensions and one spectral dimension, they require a large amount of disk space and computing power to store and analyze, but also provide a wealth of information for investigating plant disease phenotypes. Typical wavelengths observed for plant imaging experiments are the visible (400–700 nm), near-infrared (NIR; 700–1100 nm), and short-wave infrared (1100–2500 nm) regions. A major advantage of hyperspectral imaging is the wide range of measurements that can be derived from the data collected. At a more basic level, the data can be reduced to multispectral measurements, which are calculated from a few key spectral bands. For example, the normalized difference vegetation index (NDVI) is a measure of the greenness of plant tissue, calculated from reflected wavelengths in the NIR and red regions (Bauriegel and Herppich, 2014). At a more complex level, entire spectra can be analyzed with algorithms that allow for comparison of many wavelengths (Bock et al., 2010).

Different plant diseases can cause distinct spectral reflectance patterns, so hyperspectral imaging offers the potential to not only detect disease but also identify specific diseases. Mahlein et al. (2012) used hyperspectral imaging to investigate disease symptoms for three different fungal diseases of sugar beet. With adequate spatial resolution, unique patterns of reflectance in the visual and NIR ranges were sufficient to distinguish healthy sugar beet plants from plants with powdery mildew and *Cercospora* leaf spot, as well as to differentiate powdery mildew infection from *Cercospora* leaf spot infection. This approach was not successful for all of the diseases that were tested, however, as the reflectance signature of sugar beet rust infection could not be distinguished from that of healthy plants.

Hyperspectral imaging has also been used to detect head blight infection on wheat (Bauriegel et al., 2011; Bauriegel and Herppich, 2014). Currently, aerial and ground based hyperspectral imaging systems are being developed for use in agricultural fields and natural environments (Busemeyer et al., 2013; Calvino-Cancela et al., 2014). In the field, hyperspectral imaging depends on solar illumination and reflectance, so variation in environmental conditions must be accounted for in the image analysis steps. Bravo et al. (2003) used normalization methods to account for variation in illumination and reflectance, enabling the classification of yellow rust infection on wheat growing in the field. For all applications of hyperspectral imaging, extensive ground-truthing will be necessary to validate the optimal set of parameters that characterize plant disease. While further development of the hyperspectral imaging methods is needed, this technique offers great promise for phenotyping plant disease.

#### Thermal imaging

Depending on the nature of infection, pathogens have different effects on the temperature of infected plant tissues. Temperature is negatively correlated with transpiration rate (Lindenthal et al., 2005). Thus, pathogens that induce stomatal closure in plants often lead to decreased transpiration rates and increased leaf temperature. For example, Lindenthal et al. (2005) used digital infrared thermography to image downy mildew infection in cucumber, which is caused by the oomycete *Pseudoperonospora cubensis*. Infrared thermography imaging can detect relative differences in leaf surface temperature by detection of infrared radiation from leaves. Using this method, Lindenthal et al. (2005) found that different stages of infection have different effects on leaf temperature. Infection with *P. cubensis* initially causes localized decreases in surface temperature, which are thought to be due to suppression of stomata closure early during infection. At later stages when the pathogen has caused areas of necrosis, the temperature of the infected leaf tissue increases to levels that are higher than that of uninfected tissue, which may be due to the inability of damaged tissue to perform natural cooling through transpiration.

Other pathogens alter leaf surface temperature in different ways during infection. Thermal imaging of tobacco plants resistant to tobacco mosaic virus showed increases in leaf temperature prior to cell death being visible, likely due to stomata closure (Chaerle et al., 1999, 2001). The fungus *Cercospora beticola* had the opposite effect on tobacco plants, causing a decrease in temperature during infection, possibly due to the pathogen’s ability to suppress stomatal closure (Chaerle et al., 2004). Bacterial pathogens, such as *P. syringae* and *Xanthomonas campestris* pv. *campestris* suppress stomatal closure at early stages of infection to promote entry into leaf tissue (Melotto et al., 2008; Gudesblat et al., 2009), which may also lead to detectable surface temperature changes. Thus, if thermal imaging is able to detect these temperature changes caused by pathogens, it offers the potential to identify different types of plant diseases.

When used in agricultural fields, infrared thermography is sensitive to environmental variation, such as cloud cover and solar orientation (Munns et al., 2010). It can also be difficult to identify plots of interest in an image, to separate the crop canopy from soil in the background, and to adjust for different temperatures that result from different plant heights and different environmental conditions (Munns et al., 2010). Nonetheless, researchers are currently developing methods that alleviate these challenges. For example, plots are identified with signs that are readable in the thermal image, and reference surfaces are used for internal normalization of image data (Jones et al., 2009). Using these methods, different genotypes of rice and grape were distinguished based on their relative responses to drought stress. While not yet used extensively in the field specifically for biotic stresses, infrared thermography may be a useful tool as a monitoring system for general stress responses in agriculture settings, whether biotic or abiotic.

### DATA ANALYSIS

For image-based phenotyping, once the images are generated, phenotypic data must be extracted using analysis techniques. The computational methods by which these datasets are analyzed are an important consideration in any phenotyping experiment. Just as there are many options for how image data are collected, so too there are many options for how the data are analyzed.

Marr’s three levels of information processing is a helpful framework for thinking about how to apply image analysis to biological problems (Marr, 1982; Pridmore et al., 2012). According to Marr (1982), any information processing process, such as image analysis, can be divided to three levels: (1) computational theory, a description of what the process does, often in mathematical terms; (2) algorithm, the steps used by the process to implement the computational theory; and (3) mechanism, the physical systems and software that carry out the process (Pridmore et al., 2012). All three of these levels should be considered when designing a phenotyping experiment. As discussed by Pridmore et al. (2012), plant biologists may lack expertise in computer vision and often focus primarily on mechanism, relying on the software and hardware with which they are most familiar. This may not result in the best experimental design, however, so Pridmore et al. (2012) advise biologists to identify the best possible approaches based on computational theory and algorithm, and then choose the mechanism that best implements those approaches. Like so many fields, phenomics will benefit from increased multidisciplinary collaboration.

#### Image processing

In order to extract meaningful data from images, various image processing steps are necessary. Computational pipelines can be customized to carry out these steps in an automated manner. Pre-processing steps adjust for differences in lighting and alignment of the image. They may also involve conversion between different image types, such as conversion between RGB and grayscale (Klukas et al., 2014). Following pre-processing, segmentation steps can be used to partition regions of the image for selection of certain features (Klukas et al., 2014). Rousseau et al. (2013) used segmentation of *F*_v_/*F*_m_ images to assess disease symptom phenotypes caused by *Xanthomonas fuscans* subsp. *fuscans* on the common bean *Phaseolus vulgaris*. For their initial approach, areas with different stages of disease were identified with universal threshold levels set by human raters based on their visual observations of a training set of images. To improve upon this method, the authors developed a probability-based approach to identify areas of the leaf that were likely to be diseased, followed by clustering analysis to divide the diseased area into regions with different stages of disease. Additionally, the probability thresholds were normalized daily based on *F*_v_/*F*_m_ measurements from mock-inoculated controls. Overall, the probability-based approach improved the analysis both by automating the process and by accounting for day-to-day variation in *F*_v_/*F*_m_ levels.

A wide variety of algorithms can be used for classification or quantification of image features. A commonly used example for hyperspectral imaging applications is the Spectral Angle Mapper (SAM) classification algorithm (Bauriegel et al., 2011; Mahlein et al., 2012). This algorithm compares vectors representing experimentally determined spectra and reference spectra, and calculates an angle to represent the degree of difference between the two at each pixel.

A variety of available software packages can aid in development of custom image analysis approaches. ImageJ^4^ is an open source, Java-based image analysis program, which is customizable with a variety of available macros and plugins written specifically for plant phenotyping applications (Hartmann et al., 2011; Stewart and McDonald, 2014). There are also commercial software packages available for plant disease phenotyping, such as ASSESS 2.0, from the American Phytopathological Society, but these lack ability for customization. The website Plant Image Analysis^5^ provides an online database of image analysis software options for plant biology, both commercial and open source (Lobet et al., 2013). Given that image-based plant phenotyping is a relatively new field, the analysis tools are still in development.

## CHALLENGES AND FUTURE DIRECTIONS FOR IMAGE-BASED PLANT DISEASE PHENOTYPING

While image-based phenotyping methods offer great promise for enhancing characterization of plant disease phenotypes, many hurdles remain for implementing these techniques in both research and agricultural production. Since phenotyping methods enable the exploration of multiple dimensions of phenotypic space, it will be essential to determine which particular dimensions serve as the best indicators for plant disease status. As the ultimate goal is to limit the impacts of plant disease on agricultural production, understanding the correlation between the disease symptoms and impacts on yield is important. Furthermore, indicators of general stress may be effective early warning signs of disease but are not likely to identify or distinguish between different diseases. On the other hand, assays that are designed to detect specific pathogens or diseases may be more reliable but may not be useful for broad surveys or early warning systems. This tradeoff must be considered when designing phenotyping strategies.

While imaging is likely to be useful for investigating many plant–pathogen systems, there also may be cases for which imaging approaches are not sufficient for characterization of disease phenotypes. Plant diseases that progress asymptomatically or with only internal symptoms will be difficult to detect with image-based phenotyping. For example, the group of fungi known as *Fusarium* spp. cause maize ear rot disease, and certain environmental conditions promote asymptomatic *Fusarium* infections with accumulation of dangerous mycotoxins in the host tissue (Murillo-Williams and Munkvold, 2008; Mesterházy et al., 2012). Imaging approaches are unlikely to effectively diagnose this type of infection at an early stage. Instead, other non-imaging remote sensing technologies need to be developed to detect this disease or others like it. Becker et al. (2014) identified changes in a set of volatile sesquiterpenes that are emitted from corn, between plants infected with *Fusarium* and those that are uninfected. In theory, remote sensing of these different volatile chemical profiles could be a method for early detection for *Fusarium* infection. While such technologies for remote sensing do not yet exist, it may be possible to use such methods in the future to detect diseases without clear visible symptoms.

Other studies suggest that image-based phenotyping is not more accurate or sensitive than visual assessment for certain host–pathogen systems. Olmstead et al. (2001) attempted to use digital imaging to improve estimates of powdery mildew infection on sweet cherry leaves. They determined that imaging did not provide an accurate quantification of the infected leaf area, likely because the difference in color between infected and uninfected areas was not distinct enough for this particular disease (Olmstead et al., 2001). Additionally, for some applications, it may be more useful to quantify pathogen growth levels in the host rather than disease symptoms (Jackson et al., 2006). Given the wide range of infections caused by plant pathogens, the best phenotyping strategy for each particular disease should be carefully considered.

A major goal in plant disease phenotyping is to translate techniques from controlled environments in growth chambers to agricultural fields (Araus and Cairns, 2014). Many researchers are currently working on developing unmanned aerial vehicles or ground vehicles for imaging of crop canopies (Prashar et al., 2013). Using a variety of different imaging techniques simultaneously may be necessary for acquiring sufficient data to monitor plant health in the field.

Another challenge is developing hardware and software that are broadly applicable across different plant–pathogen systems. Plants vary widely in size and leaf architecture, and diseases cause different types of symptoms. Thus, most disease phenotyping methods have been developed specifically for one particular host–pathogen system. For example, imaging methods developed specifically for *A. thaliana* may not be applicable to grasses, which have a dramatically different architecture.

Moving forward, it will be necessary to standardize methods and document analysis methods for reproducibility. Making analysis software open source and available is necessary for reproducibility and will enable improvement of the phenotyping methods to be a community effort (Prlic and Procter, 2012; Leprevost et al., 2014). While many challenges remain for implementing these technologies, a multi-disciplinary approach involving collaboration between biologists, engineers, and computer scientists is the best strategy for overcoming these hurdles.

### Conflict of Interest Statement

The authors declare that the research was conducted in the absence of any commercial or financial relationships that could be construed as a potential conflict of interest.

## Footnotes

1 http://www.raspberrypi.org

2 http://www.danforthcenter.org/scientists-research/core-technologies/phenotyping

3 http://www.plantphenomics.org.au

4 http://rsbweb.nih.gov/ij

5 http://www.plant-image-analysis.org
